# Optimization of *Candida tropicalis* growth conditions on silicone elastomer material by response surface methodology

**DOI:** 10.3389/fbioe.2025.1572694

**Published:** 2025-07-17

**Authors:** Kavyasree Marabanahalli Yogendraiah, Bindu Sadanandan, Lokesh Kyathsandra Natraj, Vaniyamparambath Vijayalakshmi, Kalidas Shetty

**Affiliations:** ^1^ Department of Biotechnology, M S Ramaiah Institute of Technology, Bengaluru, Karnataka, India; ^2^ Department of Microbiological Sciences, North Dakota State University, Fargo, ND, United States

**Keywords:** *Candida tropicalis*, biofilm, optimization, response surface methodology, central composite design, Johnson transformation

## Abstract

Biofilm in the emerging pathogen *Candida tropicalis* and the most prevalent Non-*Albicans Candida* infections is linked to fouling of medical devices and virulence. The growth conditions (temperature, media pH, incubation time, inoculum size, and shaker speed) for clinical cultures of *C. tropicalis* were optimized on silicone elastomer material by Central composite design based on Response surface methodology. Six clinical cultures (C4, U873, U951, U1179, U1309 and U1360) and a standard culture (MTCC-184) were chosen for the study. Growth and biofilm were quantified for all the cultures by crystal violet (biofilm), MTT (cell viability), calcofluor white (cell mass), and wet and dry weight (cell mass) measurements. Among the isolates, U951 was found to fit the CCD model. The non-normal distribution and heteroscedasticity of the data favored the transformation via CCD-integrated Johnson model profiler for the prediction of the optimal growth conditions. For U951 isolate, biofilm formation was impacted by temperature and incubation time. A direct correlation was observed between biofilm formation and cell viability, with variations in the cell mass in all the cultures. This is the first of its kind study to advance an *in vitro* silicone elastomer-based high-throughput growth model of *C. tropicalis* for various applications, including the screening of potential therapeutics.

## 1 Introduction

*Candida* species are opportunistic pathogens that cause significant risks to public health as they are a leading source of morbidity and mortality across the globe (Santos et al., 2018). Currently, *Candida* spp., is the third most frequent cause of bloodstream infections, including candidemia (Chen et al., 2021). At least 15 different species of *Candida* can cause disease in humans, however, only five of these pathogens are primarily responsible for invasive infections: *Candida albicans, Candida tropicalis, Candida glabrata (Nakaseomyces glabrata), Candida parapsilosis, and Candida auris (Candidozyma auris)* (Pappas et al., 2018; Lass-Flörl et al., 2024). Amongst the five species, *C*. *albicans* is the most common *Candida* pathogen (Sadanandan et al., 2023; Palchaudhuri and Chattopadhyay, 2019). *C. tropicalis* is included as the “highest priority” fungal pathogen in the recent World Health Organization (WHO) list, being the most dangerous fungal pathogen causing infections (Zhang et al., 2020; Sharma and Chakrabarti, 2023). According to a global multicentre surveillance survey, *C. tropicalis* accounts for about 9% of all *Candida* cases. However, it is more common in the Asia-Pacific region, where it accounts for 14% of the cases, and in India, it is 41%. Invasive *C. tropicalis* infections have a death rate that can range from 61% to 77.8%, which is much greater than that of other *Candida* species (Fan et al., 2023). Epidemiological data show that both men and women are equally susceptible to *C. tropicalis*-caused candidemia as compared with other species of *Candida* (Negri et al., 2011; Soulountsi et al., 2021). Additionally, *C. tropicalis* is linked to higher mortality and dissemination potential in ICU patients, especially those with cancer, than any other non-*albicans Candida* (NAC) species or *C. albicans* (Yang et al., 2023).

It has been observed that *C. tropicalis* is a highly potent biofilm former, outperforming *C. albicans* in the majority of the studies (Zuza-Alves et al., 2017; Lima et al., 2022; de Souza et al., 2023). *Candida* biofilms are complex microbial communities that can adhere to a variety of surfaces, both living and non-living surfaces (Malinovská et al., 2023). The development of biofilms by *C. albicans* and other non-*albicans* species has been linked to the pathogenicity of persistent and recurring infections. These biofilms are strongly associated with drug resistance and virulence because they cause biofouling of medical devices, which makes clinical therapy more challenging (Tobouti et al., 2016; Sultan, 2019; Tseng et al., 2020; Van Genechten et al., 2024). Silicone elastomers are widely used in medical devices and point-of-care (POC) diagnostics due to their hydrophobicity, elasticity, biocompatibility, and chemical resistance. However, their surfaces are prone to *Candida* colonization, leading to biofilm formation, biofouling, and serious infections (Zare et al., 2021).

Optimization and modeling are essential for improving process efficiency and cost-effectiveness. The traditional one variable at a time (OVAT) method is time-consuming and fails to analyze interactions between variables (Venkatachalam et al., 2021). Central Composite Design (CCD)-based RSM offers a more efficient alternative by reducing experimental runs while enabling robust optimization (Sadanandan et al., 2021). A group of statistical techniques known as RSM was used to build models, design trials, and simultaneously evaluate the effects of different factors and establish optimal conditions. The link between a set of quantitative experimental conditions and one or more response variables is examined using CCD-based RSM (Almeida et al., 2017). Some of the studies employing RSM and soil cultures of *C. tropicalis* that find wide industrial applications have been reported previously (He and Tan, 2006; Ling et al., 2011; Almeida et al., 2017; Madian et al., 2019; Thangavelu et al., 2021). Among the various examples are valuable products such as lipase enzyme, biosurfactants, biodiesel, xylitol, and bioethanol (Alarifi et al., 2023).

RSM was used to optimize culture conditions for *C. tropicalis* clinical isolates. However, its predictive power is limited by the organism’s adaptive responses. To improve accuracy, a Johnson-based transformation was applied to normalize skewed data, enabling more reliable optimization results. In experiments with multiple samples, some data naturally deviate from significance and follow a skewed distribution. Normalizing this skew reduces statistical errors and data variability. Among various transformation models, the Johnson transformation effectively converts non-normal data into a normal distribution while preserving key characteristics and linearizing relationships (Liu, 2009; Cai and Xu, 2024).

This study employs the optimization of *C. tropicalis* growth conditions on silicone elastomer material in 96-well microtiter plates by using CCD-based RSM and CCD integrated model profiler, i.e., Johnson transformation, which was used to obtain statistically reliable optimized growth conditions for culturing of clinical cultures of *C. tropicalis*. The Johnson transformation was applied to transform datasets to follow a normal distribution. Temperature, pH of the media, shaker speed, inoculum size, and incubation time were considered to determine if the parameters affected the growth of *C. tropicalis* cultures independently or interactively. Crystal violet (CV) assay, MTT assay, Calcofluor white assay, wet and dry weight methods were also employed to quantify the biofilm as a means of comparing cell viability, cell mass, and biofilm formation. This is one of the first of its kind approaches designed and validated for the optimization of *C. tropicalis* growth conditions on silicone elastomer material by using CCD-based RSM. The innovative integration of CCD and JT for optimizing *C. tropicalis* growth conditions on silicone elastomer, a substrate widely used in medical devices, shows promise in the optimization of dynamic systems such as microbial growth and biofilm formation on engineered biomaterials.

## 2 Methodology

### 2.1 Cultures, growth media, and growth conditions

A total of six distinct *C. tropicalis* clinical cultures from urine (U873, U951, U1179, U1309, U1360) and sputum (C4) samples of both male and female patients were used for the study. These cultures were isolated from individuals who had invasive candidiasis and were provided by the Microbiology Laboratory at M S Ramaiah Medical College and Teaching Hospital in Bengaluru, India. These clinical cultures have been identified by the VITEK system along with the Antibiotic sensitivity test (AST), at the Department of Neuromicrobiology, NIMHANS, Bengaluru, India. A standard *C. tropicalis* MTCC-184 (ATCC-750), a blood isolate, was procured from IMTECH, Chandigarh, India. Of the six clinical isolates, four (C4, U873, U1179 and U1360) have been whole genome sequenced and the data is available on the NCBI SRA portal (https://www.ncbi.nlm.nih.gov/sra/SRX11881291[accn] (Sadanandan and Manivelan, 2021). The study was conducted with clinical cultures and involved no human or animal participants directly; hence, ethical clearance was not required.

The identity of the cultures was reconfirmed in our laboratory based on the colony colour produced on CHROMagar media. The biofilm tube test was carried out to screen for biofilm formation. *C. tropicalis* cultures were grown in Tryptic Soy Broth (TSB) and incubated for 24 h at 37°C in an incubator shaker at 100 rpm for biofilm formation and optimization studies. Glycerol stocks were maintained at −86°C.

### 2.2 Substrate material

Silicone elastomer material was locally sourced from Royal Industrial Stores, Bengaluru, India. The properties of the material, as per the test certificate provided by the vendor, are included in (Supplementary Table S2). It was used as the abiotic substrate material for biofilm formation. It was cut into discs of 6 mm diameter and placed in sterile 96-well microtiter plates (Axiva Volex). Silicone elastomer was chosen as the substrate material as it has far-reaching applications in medical devices, especially catheters, filler material, and entire implants, or as a covering material (De-la-Pinta et al., 2019; Lafuente-Ibáñez de Mendoza et al., 2021).

### 2.3 RSM for optimizing the growth conditions of *Candida tropicalis* biofilm

Different parameters such as temperature (26.5, 30, 33.5, 37, 40.5°C), media pH (5.4, 6.0, 6.6, 7.2, 7.8), shaker speed (52.5, 97.5, 142.5 rpm), inoculum size (1.5%, 6%, 10.5%, 15%, 19.5%) and incubation time (12, 24, 36, 48, 60 h) were chosen to optimize the growth of *C. tropicalis* biofilm formation on silicone elastomer discs in 96-well microtiter plates. The range of these parameters was chosen based on previously published reports (THEIN et al., 2007; Deorukhkar et al., 2014; Rezazadeh et al., 2016; Santos et al., 2016; Salmanizadeh et al., 2024). Temperature was indicated with the number (1), media pH (2), shaker speed (3), inoculum size (4), and incubation time (5). A total of 27 sets of experiments were obtained for the optimization of growth conditions of *C. tropicalis* biofilm by CCD -based RSM. Data analysis was conducted using StatSoft^®^ STASTICA version 12.6 statistical software.

### 2.4 Pre-inoculum preparation

Pre-inoculum was prepared by inoculating a loopful of culture into 5 mL of Tryptic soy broth (TSB). This inoculum was incubated overnight at 37°C. Optical density was measured using a UV spectrophotometer at 600 nm. *C. tropicalis* cells were enumerated using a haemocytometer to adjust the cell concentrations to 1 × 10^6^ cells mL^−1^ and used as stock for further experiments.

### 2.5 Induction of *Candida tropicalis* biofilm on silicone elastomer material

Silicone elastomer material discs were placed in 96-well microtiter plates to induce *C. tropicalis* biofilm formation. The optical density of *C. tropicalis* suspension was determined for each culture in TSB media at 600 nm. These cells were then enumerated using a haemocytometer, and cell suspension at a concentration of 1 × 10^6^ cells mL^−1^ was used as the stock. A volume of 100 μL of each suspension was added on silicone elastomer discs and incubated at 37°C for 90 min in an incubator shaker for adhesion. Following incubation, the wells were washed twice with 1× phosphate-buffer saline (PBS) to remove non-adherent cells. Fresh media of pH 5.4, 6.0, 6.6, 7.2 and 7.8 was added to the wells containing the discs and the plates were incubated at temperatures of 26.5, 30, 33.5, 37°C and 40.5°C at growth periods of 12, 24, 36, 48 and 60 h in an incubator shaker at speeds of 52.5, 97.5 and 142.5 rpm. For each of these culture conditions, the experiment is performed individually on the silicone elastomer material and thereafter quantified using the CV assay at 570 nm (Sadanandan et al., 2021). A second-order regression model was used to assess the experimental results of the study, and the following equation was deduced based on the coefficient values of each identified constituent as shown in Equation 1.

$$\begin{array}{ll} Y= & \beta_{0}+\beta_{1}X_{1}+\beta_{2}X_{2}+\beta_{3}X_{3}+\beta_{4}X_{4}+\beta_{5}X_{5}+\beta_{11}X_{1}^{2}+\beta_{22}X_{2}^{2}\\ & +\beta_{33}X_{3}^{2}+\beta_{44}X_{4}^{2}+\beta_{55}X_{5}^{2}+\beta_{12}X_{1}X_{2}+\beta_{13}X_{1}X_{3}+\beta_{15}X_{1}X_{5}\\ & +\beta_{23}X_{2}X_{3}+\beta_{24}X_{2}X_{4}+\beta_{25}X_{2}X_{5}+\beta_{34}X_{3}X_{4}+\beta_{35}X_{3}X_{5}\\ & +\beta_{45}X_{4}X_{5} \end{array}$$ (1)

where, Y is the response, β_0_ is the intercept, X_1_, X_2_, X_3_, X_4_, X_5_ are coded values for different parameters, β_1_, β_2_, β_3_, β_4_, β_5_, are coefficients estimated by the model for linear effects; β_11_, β_22_, β_33_, β_44_, β_55_, are quadratic effects and β_12_, β_13_, β_15_, β_23_, β_24_, β_25_, β_34_, β_35_, β_45_ are interactive effects of the growth parameters under consideration. Furthermore, for the significant culture that fit the model, the ANOVA (analysis of variance), Pareto chart, contour plot, and desirability plot were analyzed.

#### 2.5.1 Validation of the experiment by CV assay

Validation was carried out by repeating the experiment on silicone elastomer material for the optimal conditions given by the CCD based RSM model by CV assay. A comparison was made between the predicted and observed CV values.

### 2.6 CCD integrated model profiler (Johnson transformation)

The CCD integrated model profiler-Johnson transformation (CCD-JT) was used for the optimization of growth conditions for the non-statistically significant *C. tropicalis* cultures and for the dependent variables, which did not follow normal distribution. Based on the optimal conditions given by CCD-JT, a capability analysis test was performed, which in turn gave the predictive value/target value and then compared with the observed value by performing a CV assay to check if there was any significant difference between the observed and predicted values.

#### 2.6.1 Validation of CCD integrated model profiler (Johnson transformation)

The experimental validation was done by performing a CV assay under the optimal conditions provided by the CCD integrated model profiler (Johnson transformation). The assay was performed in triplicates to compare the observed values with those of the predicted values.

### 2.7 Biofilm quantification of *Candida tropicalis* cultures with their optimized conditions

*C*. *tropicalis* biofilm formed on 96-well microtiter plates was quantified by different methods: CV, MTT and Calcofluor assays, along with wet weight and dry weight methods. All the experiments were performed in triplicates.

#### 2.7.1 CV assay

CV assay was performed to assess the biofilm formation in *C. tropicalis* cultures. Following the incubation period, the medium was discarded, and the wells were rinsed twice with PBS. Subsequently, 1% CV stain was added to each well and incubated for 20 min at 37°C and then the CV staining solution was discarded. Following this a volume of 150 μL of 95% ethanol was added, and the absorbance of each well was measured at 570 nm using an automated microplate reader (Sadanandan et al., 2021; Li et al., 2003).

#### 2.7.2 MTT assay

MTT assay was performed to determine cell viability, as per the previously published protocol (Contreras Martínez et al., 2022). Upon biofilm formation at optimized conditions, the media containing the cells were discarded, and the wells were washed with PBS. MTT (5 mgmL^−1^) was added to each well, and incubated at 37°C for 3 h in dark. After the incubation period, acidified isopropanol (IPA) was added to dissolve the formazan crystals. The absorbance was then measured spectrophotometrically using a multimode microplate reader (Synergy HT, Biotek) at 540 nm.

#### 2.7.3 Calcofluor white binding assay

To determine *C. tropicalis* biofilm cell mass calcofluor white assay was performed. This assay was based on a modified protocol from (Punjabi et al., 2020). Following the incubation period, the medium was discarded, and then 150 μL of Calcofluor white stain was added to each well and incubated in dark at room temperature for 45 min. Later the staining solution was removed and 100 μL of Tris-EDTA (TE) buffer (10 mM Tris-HCl, pH 8.0, 10 mM EDTA) was added and the fluorescence was measured at excitation/emission of 360/460 nm using a multimode microplate reader (Synergy HT, Biotek).

#### 2.7.4 Wet weight and dry weight measurements

Gravimetric measurements of the wet and dry weights of *C. tropicalis* cultures were performed, with slight modifications to (Tseng et al., 2020). The weight of an empty 96-well plate was recorded and the cultures were grown at the desired optimal growth conditions. The weight of the plate without media was recorded. The difference in the weight of the microplate with media and the empty plate gives the wet weight. For dry weight, the growth media was discarded and the plate was dried at 60°C for 1 h. The difference in the weight of the dried plate and the empty plate provides the dry weight of the biofilm.

### 2.8 Statistics

Two-way ANOVA followed by Tukey’s multiple comparison test was performed for the CV assay to study any statistical significance between predicted and observed values given by the conventional CCD and the CCD-JT. All the assays were performed in triplicates.

## 3 Results

### 3.1 Identification and screening of cultures for biofilm formation

Based on the identification by CHROMAgar *Candida* test, dark blue colony colour was observed for *C. tropicalis* MTCC-184, C4, U873, U951, U1179, U1309, and U1360 after 96 h of incubation at 37°C on HiCrome™agar medium, as shown in (Supplementary Figure S1). For biofilm formation, biofilm tube test was carried out where some of the *C. tropicalis* cultures showed a dark ring of CV stain around the test tubes and were categorized as high biofilm formers (MTCC-184, C4, U873) intermediate biofilm formers (U951, U1360) and low biofilm formers (U1179, U1309) as shown in (Supplementary Figure S2). We have used three different categories of *C. tropicalis* clinical strains isolated from patient blood, urine and sputum. Such isolates are commonly associated with biofilm-related infections, such as urinary tract infections and respiratory conditions, and thus serve as relevant models for studying biofilm formation in clinical contexts.

### 3.2 Optimization of *Candida tropicalis* growth conditions for biofilm formation

**FIGURE 1:**
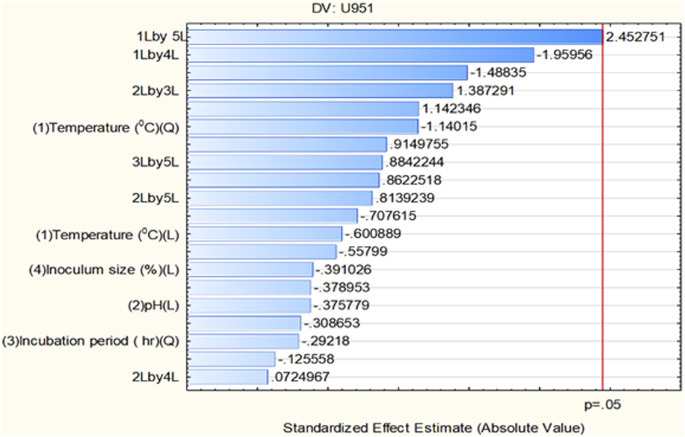
Pareto chart showing significant independent factor(s) at linear (L) and quadratic (Q) form for the *Candida tropicalis* U951 isolate grown on silicone elastomer material.

**FIGURE 2:**
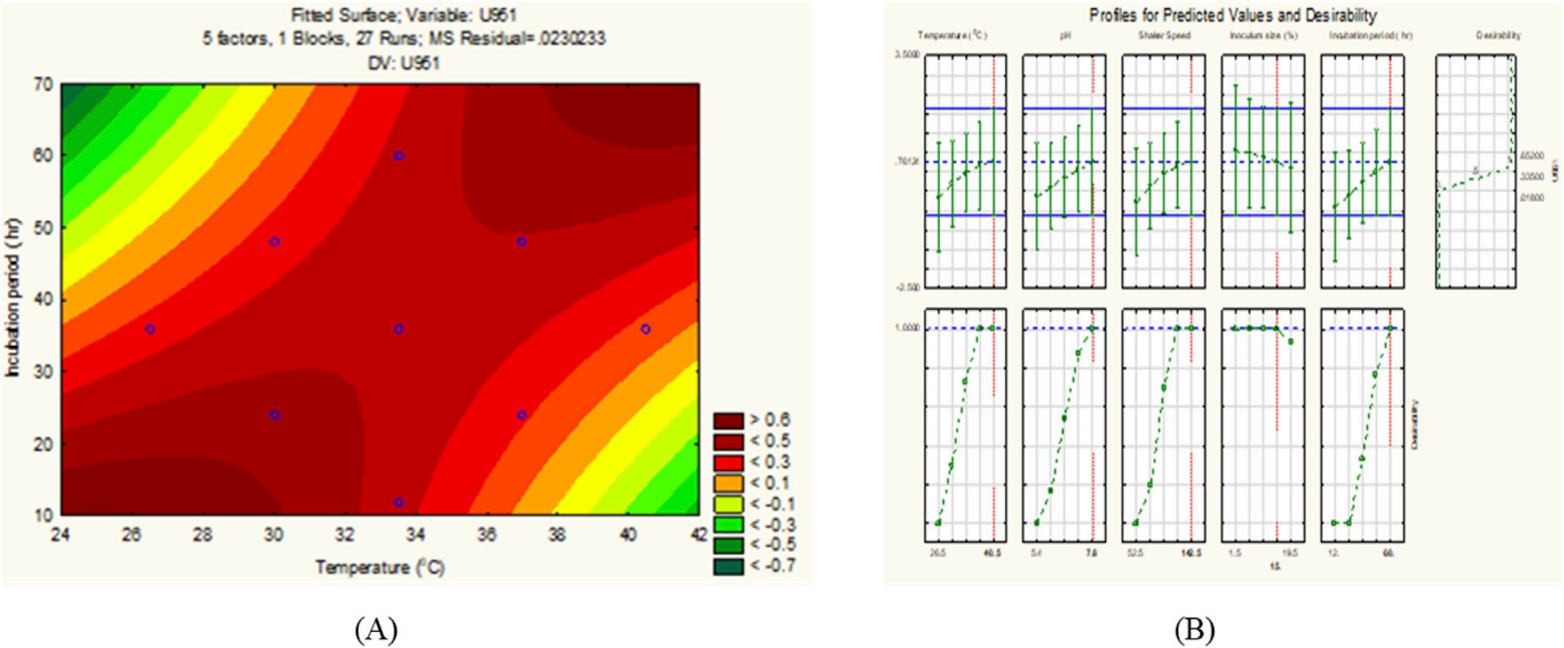
**(A)** 2D contour response surface plot, and **(B)** Desirability plot of *C*. *tropicalis* U951 grown on silicone elastomer material.

All seven *C. tropicalis* cultures were subjected to the growth conditions listed in Table 1. The pre-inoculum stock was used to prepare different inoculum sizes of 1.5, 6, 10.5, 15, and 19.5%. For each of the inoculum size percentages (1.5, 6, 10.5, 15, and 19.5%), growth OD and cell count were determined as shown in Supplementary Figures S4, S5 and Supplementary Tables S2, S3. For the optimization study, a CV assay was performed for all the cultures to observe biofilm formation. For the U951 isolate, the optimized conditions given by the CCD-based RSM model are: Temperature of 32.7°C, pH- 7.14, Incubation time- 38.5 h, Inoculum size – 14% and Shaker speed- 110 rpm. It was found that only the U951 culture was statistically significant when grown on silicone elastomer material and fits the CCD model. The CCD model tested the linear (L) as well as quadratic (Q) forms of interaction effects of the growth variables. The 1L by 5L refers to the interaction between temperature (1L) and incubation period (5L) which were found to be the significant parameters for U951 culture, as they show p = 0.05 vertical line in the pareto chart as shown in Figure 1, whereas other cultures were found to be non-significant as shown in Supplementary Figure S3. The p-value serves as a useful tool for assessing the importance of each of the coefficients. Plotting contour plots (Figure 2) gives us the saddle point, which allows us to determine the interaction effects and optimal levels of the variables. The analysis of variance (ANOVA) revealed significant independent factors for U951 based on the F and p values indicated in Table 2. The predicted solution for CCD-based RSM was calculated, and the second-degree polynomial regression equation was derived for U951 as shown in Equation 2.`

$$\begin{array}{ll} Y_{U951\;}= & 0.37-0.37x_{1}-0.08x_{1}^{2}-0.02x_{2}-0.02x_{2}^{2}-0.04x_{3}-0.11x_{3}^{2}\\ & -0.02x_{4}-0.03x_{4}^{2}-0.01x_{5}-0.22x_{5}^{2}\;0.14x_{1}x_{4}+0.105x_{2}x_{3} \end{array}$$ (2)

where Y = CV value for the biofilm formed, x_1_ = Temperature, x_2_ = pH, x_3_ = incubation period, x_4_ = inoculum size, x_5_ = shaker speed.

**TABLE 1:** RSM Design Matrix and CV assay response of *Candida tropicalis* on silicone elastomer material.

Run number	Temperature (°C)	pH	Shaker speed (rpm)	Inoculum size (%)	Incubation period (h)	MTCC-184	C4	U873	U951	U1179	U1309	U1360
1	30.0	6.0	75.0	6.0	48.0	0.191333	0.222000	0.389000	0.210333	0.106000	0.072333	0.042667
2	30.0	6.0	75.0	15.0	24.0	0.570000	0.688000	0.624667	0.652000	0.346667	0.174333	0.287333
3	30.0	6.0	120.0	6.0	24.0	0.439000	0.182667	0.170000	0.174000	0.241000	0.014333	0.040667
4	30.0	6.0	120.0	15.0	48.0	0.405000	0.188333	0.678667	0.089000	0.100667	0.209667	0.079667
5	30.0	7.2	75.0	6.0	24.0	0.231000	0.430333	0.576333	0.335667	0.147667	0.029667	0.132667
6	30.0	7.2	75.0	15.0	48.0	0.304333	0.369667	0.308333	0.077667	0.251667	0.272333	0.081667
7	30.0	7.2	120.0	6.0	48.0	0.415333	0.160667	0.281333	0.018000	0.174667	0.114000	0.124333
8	30.0	7.2	120.0	15.0	24.0	0.304667	0.053333	0.647333	0.386333	0.440000	0.265333	0.344000
9	37.0	6.0	75.0	6.0	24.0	0.464333	0.523000	0.568000	0.388667	0.456667	0.314333	0.389667
10	37.0	6.0	75.0	15.0	48.0	0.479000	0.419000	0.571000	0.071000	0.069333	0.051333	0.064333
11	37.0	6.0	120.0	6.0	48.0	0.309667	0.267667	0.309333	0.248000	0.156333	0.209333	0.291000
12	37.0	6.0	120.0	15.0	24.0	0.252000	0.391000	0.305000	0.059333	0.265667	0.213667	0.294000
13	37.0	7.2	75.0	6.0	48.0	0.287667	0.527667	0.495667	0.391000	0.311667	0.194333	0.320000
14	37.0	7.2	75.0	15.0	24.0	0.244667	0.132333	0.362000	0.050667	0.115333	0.356333	0.317000
15	37.0	7.2	120.0	6.0	24.0	0.249333	0.252000	0.284333	0.208333	0.168000	0.327333	0.088000
16	37.0	7.2	120.0	15.0	48.0	0.273000	0.253000	0.385667	0.332667	0.237667	0.274667	0.341667
17	26.5	6.6	97.5	10.5	36.0	0.186000	0.263333	0.280667	0.291000	0.119333	0.124000	0.309333
18	40.5	6.6	97.5	10.5	36.0	0.214667	0.594000	0.667333	0.164333	0.219667	0.146000	0.240333
19	33.5	5.4	97.5	10.5	36.0	0.410667	0.498667	0.790333	0.400667	0.525000	0.536667	0.378667
20	33.5	7.8	97.5	10.5	36.0	0.646000	0.638000	0.684667	0.307000	0.376667	0.269667	0.510667
21	33.5	6.6	52.5	10.5	36.0	0.261667	0.227000	0.429000	0.141000	0.307000	0.185000	0.218667
22	33.5	6.6	142.5	10.5	36.0	0.343330	0.380333	0.291333	0.208667	0.086664	0.163667	0.121000
23	33.5	6.6	97.5	1.5	36.0	0.231333	0.333000	0.470667	0.352000	0.296333	0.046000	0.202333
24	33.5	6.6	97.5	19.5	36.0	0.511000	0.328333	0.376000	0.334333	0.273000	0.323000	0.342333
25	33.5	6.6	97.5	10.5	12.0	0.467000	0.231333	0.227000	0.175333	0.128000	0.069333	0.332333
26	33.5	6.6	97.5	10.5	60.0	0.527333	0.586000	0.594000	0.537333	0.302333	0.192333	0.188000
27	33.5	6.6	97.5	10.5	36.0	0.244333	0.189333	0.225000	0.334667	0.168667	0.143000	0.204667

**TABLE 2:** ANOVA (Analysis of variance) analysis of various growth parameters under consideration for *Candida tropicalis* U951.

Factors	ANOVA; Var.: U951; R-sqr = 0.77726; Adj:.0348 (1) 5 factors, 1 blocks, 27 runs; MS Residual = 0.0230233 DV: U951				
	SS	df	MS	F	p
(1) Temperature (°C) (L)	0.008313	1	0.008313	0.361068	0.569902
Temperature (°C) (Q)	0.029929	1	0.029929	1.299946	0.297682
(2) pH (L)	0.003251	1	0.003251	0.141210	0.720009
pH (Q)	0.002193	1	0.002193	0.095267	0.768022
(3) Shaker Speed(L)	0.011528	1	0.011528	0.500719	0.505728
Shaker Speed(Q)	0.051001	1	0.051001	2.215173	0.187229
(4) Inoculum size (%) (L)	0.003520	1	0.003520	0.152902	0.709285
Inoculum size (%) (Q)	0.003306	1	0.003306	0.143606	0.717770
(5) Incubation period (h) (L)	0.000363	1	0.000363	0.015765	0.904183
Incubation period (h) (Q)	0.001965	1	0.001965	0.085369	0.779989
1L by 2L	0.017117	1	0.017117	0.743478	0.421669
1L by 3L	0.019275	1	0.019275	0.837180	0.395487
1L by 4L	0.088407	1	0.088407	3.839891	0.097754
1L by 5L	0.138508	1	0.138508	6.015990	0.049605
2L by 3L	0.044310	1	0.044310	1.924576	0.214687
2L by 4L	0.000121	1	0.000121	0.005256	0.944563
2L by 5L	0.015252	1	0.015252	0.662472	0.446779
3L by 4L	0.030044	1	0.030044	1.304956	0.296838
3L by 5L	0.018001	1	0.018001	0.781853	0.410604
4L by 5L	0.007168	1	0.007168	0.311353	0.597040
Error	0.138140	6	0.023023		
Total SS	0.620190	26			

Abbreviations indicate SS-sum, of squares; d*f*-degree of freedom; MS-mean square; F-F value; p-p value (0.05); L-linear; Q-quadratic.

#### 3.2.1 Validation of experiment

**FIGURE 3:**
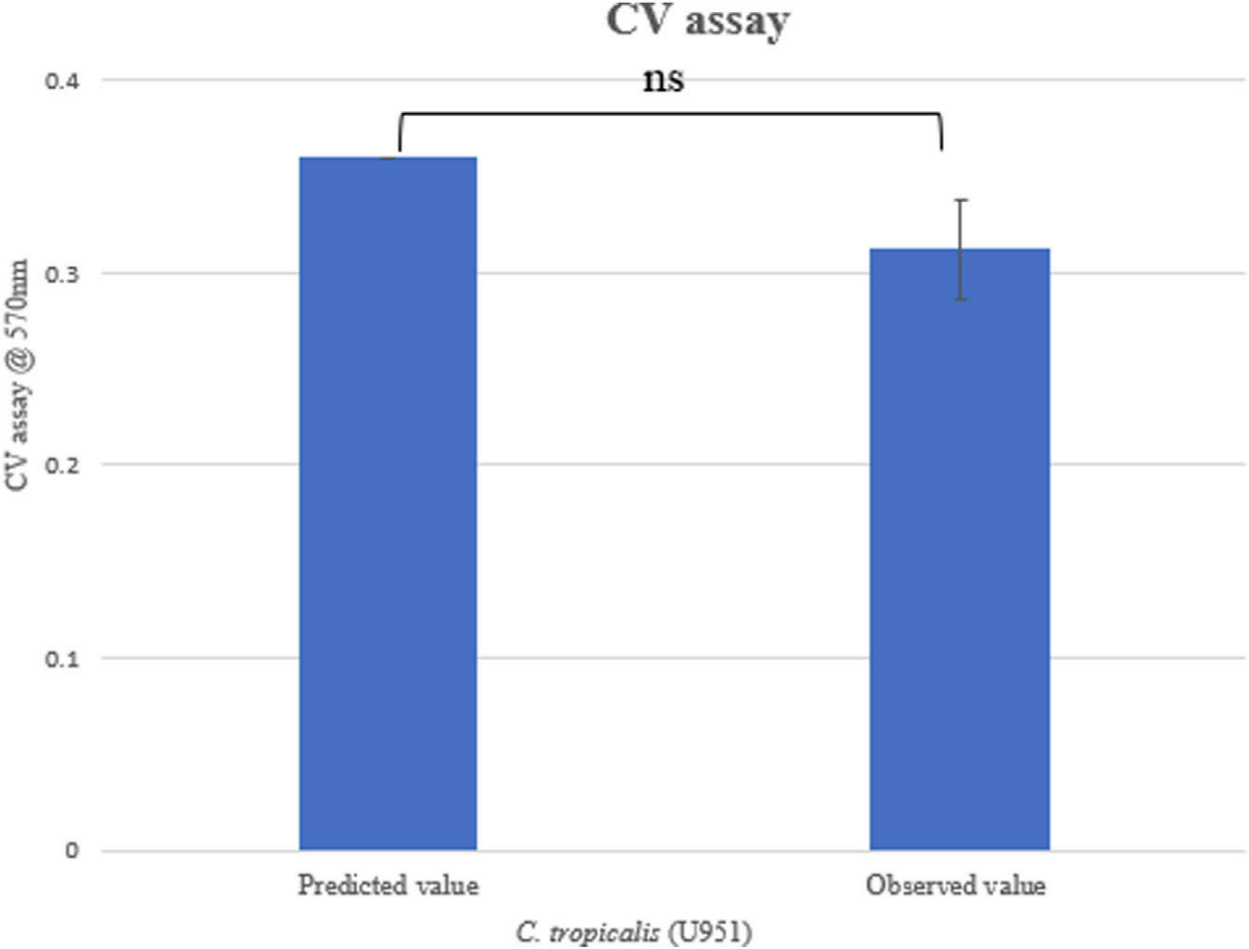
Validation of CCD model for *Candida tropicalis* U951 by CV assay.

For validating the optimal conditions of *C*. *tropicalis* U951, the culture was grown on silicone elastomer material, and the CV assay was performed at 570 nm, as shown in Table 3. The observed value was found to be close to the predicted value (Figure 3), and based on the statistical analysis, it was observed that there was no significant difference between the observed and the predicted values of the CCD-based RSM. As MTCC-184, C4, U873, U1179, U1309, and U1360 did not fit the CCD model, they were further investigated using Johnson transformation studies to obtain the best growth parameters.

**TABLE 3:** Optimized growth conditions for *Candida tropicalis* U951.

Culture	Temperature (°C)	pH	Shaker speed (rpm)	Inoculum size (%)	Incubation time (h)	Predicted value	Observed value (mean ± SD)
U951	32.7	7.14	110	14	38.5	0.36	0.312 ± 0.026

### 3.3 CCD integrated model profiler (Johnson transformation)

In the CCD model analysis, we observed that the *C. tropicalis* cultures MTCC-184, C4, U873, U1179, U1309, and U1360 did not fit the model as the ANOVA data interpretation based on p-value and F-value were found to be statistically insignificant and failed to cross the standardized effect reference value of p = 0.05 in the pareto chart analysis. The conventional CCD models reportedly have several issues, such as chances of lack of fit, which occurs when the observed error is low as compared to the residual error. In such cases, CCD yields useful, statistically reliable information via integrating a mathematical transfer function, which works by removing outlier functions and obtaining normally distributed transformed datasets (Cherevko, 2023).

To retrieve significant and reliable statistical outcomes, there are various distribution methods such as Rayleigh, Weibull, Gaussian, Johnson, and Bernoulli transformation. The quality of transformed data was analyzed based on Lilliefors (Kolmogorov-Smirnov probability distribution test), where D_α_ = 0.565 (D < D_α_ to prove the null hypothesis). In the present investigation, we observed that the datasets of *C. tropicalis* cultures MTCC-184, C4, U873, U1179, U1309, and U1309 followed the K-S d test criteria. The p-value for the *C. tropicalis* cultures as per the K-S d test of the CCD-JT is presented in Table 4. After the quality analysis of the model, the input datasets of the CCD-JT provided optimum growth conditions. Further, the predicted solution was validated by using a capability test, which is a type of process capability analysis that provides a predictive/target value. The experiments were performed at optimum conditions as suggested by the CCD model profiler and compared with the target prediction. CCD model profiler (Johnson transformation) predicted one unified optimal condition that is, temperature of 35°C, pH of 6.6, shaker speed of 38 rpm, inoculum size of 7% and incubation time of 45 h, but the capability analysis has predicted different target values for each of the cultures which were experimentally confirmed and reported as observed value with standard deviation (Table 4).

**TABLE 4:** Validation of CCD-JT condition by CV assay.

*C*. *tropicalis* cultures	Predicted CCD-JT optimized growth conditions	K-S d test*	Capability test predicted value	Observed value (CV assay)
MTCC-184	Temperature = 35°C pH = 6.60 Shaker speed = 38 rpm Inoculum size = 7% Incubation time = 45 h	0.062	0.34	0.312 ± 0.026
C4		0.048	0.34	0.318 ± 0.003
U873		0.076	0.42	0.445 ± 0.008
U1179		0.061	0.22	0.233 ± 0.026
U1309		0.055	0.19	0.156 ± 0.007
U1360		0.082	0.25	0.233 ± 0.026

K-S d test*: Lilliefors (Kolmogorov-Smirnov) probability distribution test, where D_α_ = 0.565 (D < D_α_ to prove null hypothesis).

#### 3.3.1 Validation of experiment

**FIGURE 4:**
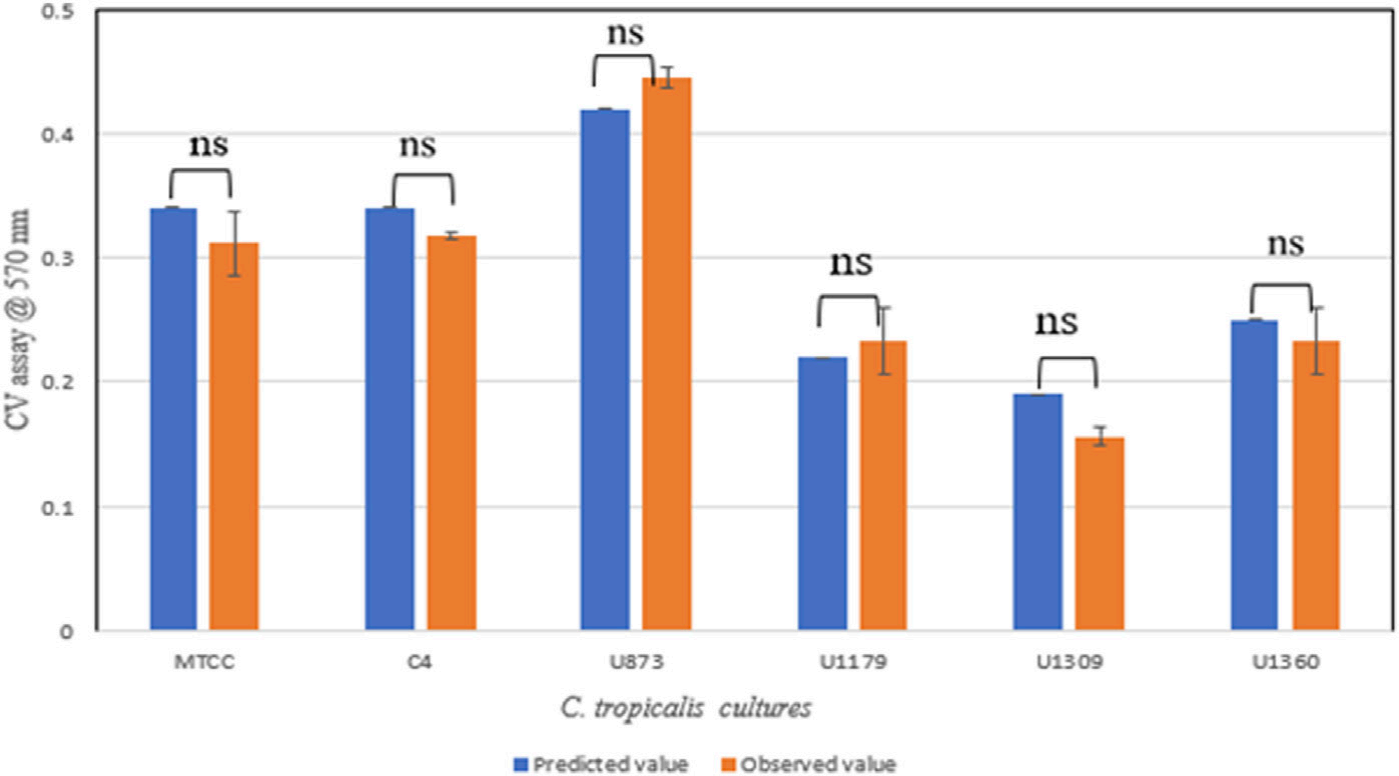
Validation of CCD-JT for *Candida tropicalis* cultures by CV assay.

Validation of the experiment by CV assay was performed for the optimized conditions given by CCD-JT (Table 4). The observed value was found to be close to the predicted value and based on the statistical analysis, it was observed that there was no significant difference between the observed and predicted values (Figure 4).

**FIGURE 5:**
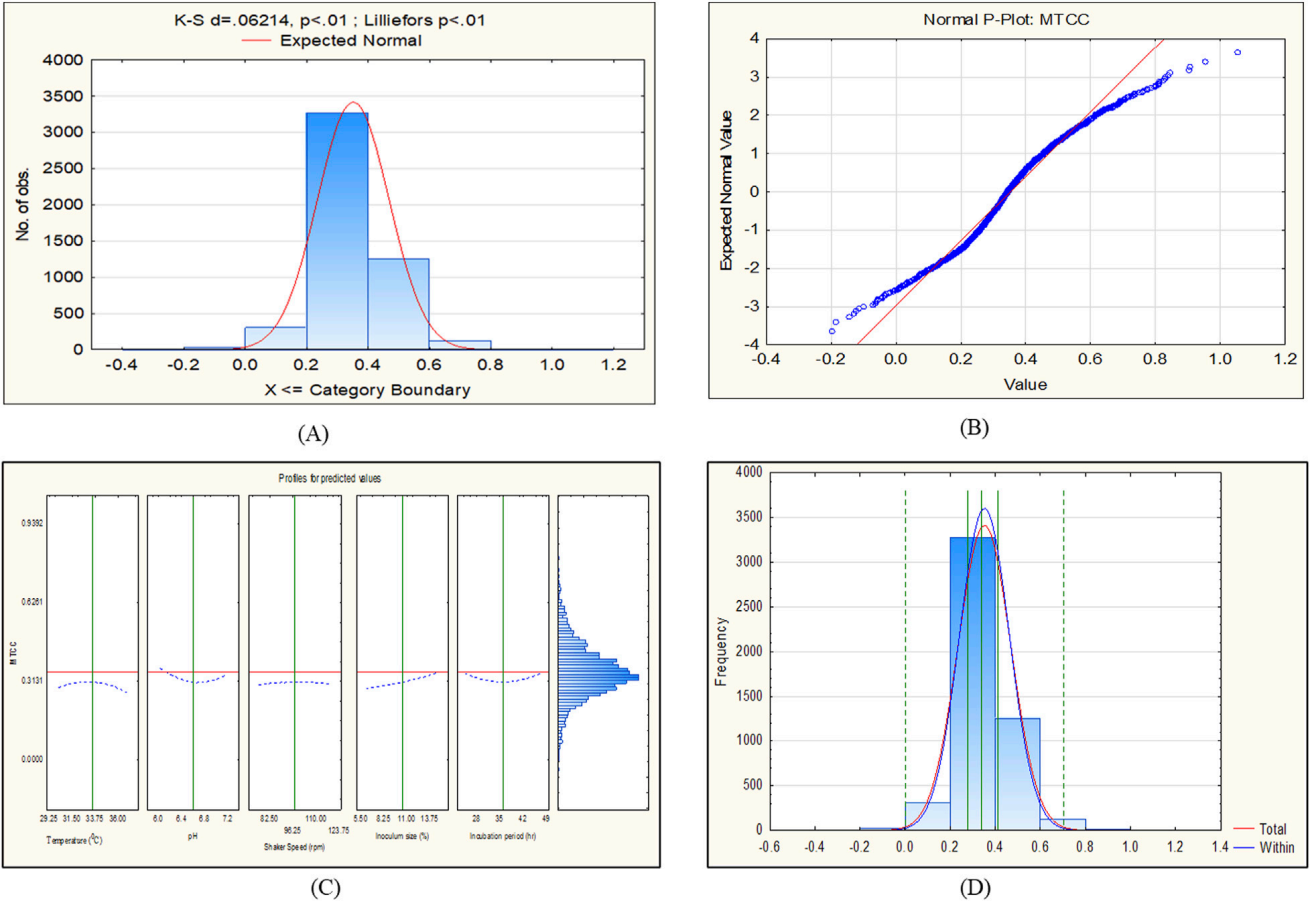
CCD-JT analysis for MTCC-184. **(A)** p-value for normal distribution based on Lilliefors test, **(B)** Fitted normal distribution profile for predicted and observed values, **(C)** Predictive optimization of growth conditions, **(D)** Capability test analysis.

**FIGURE 6:**
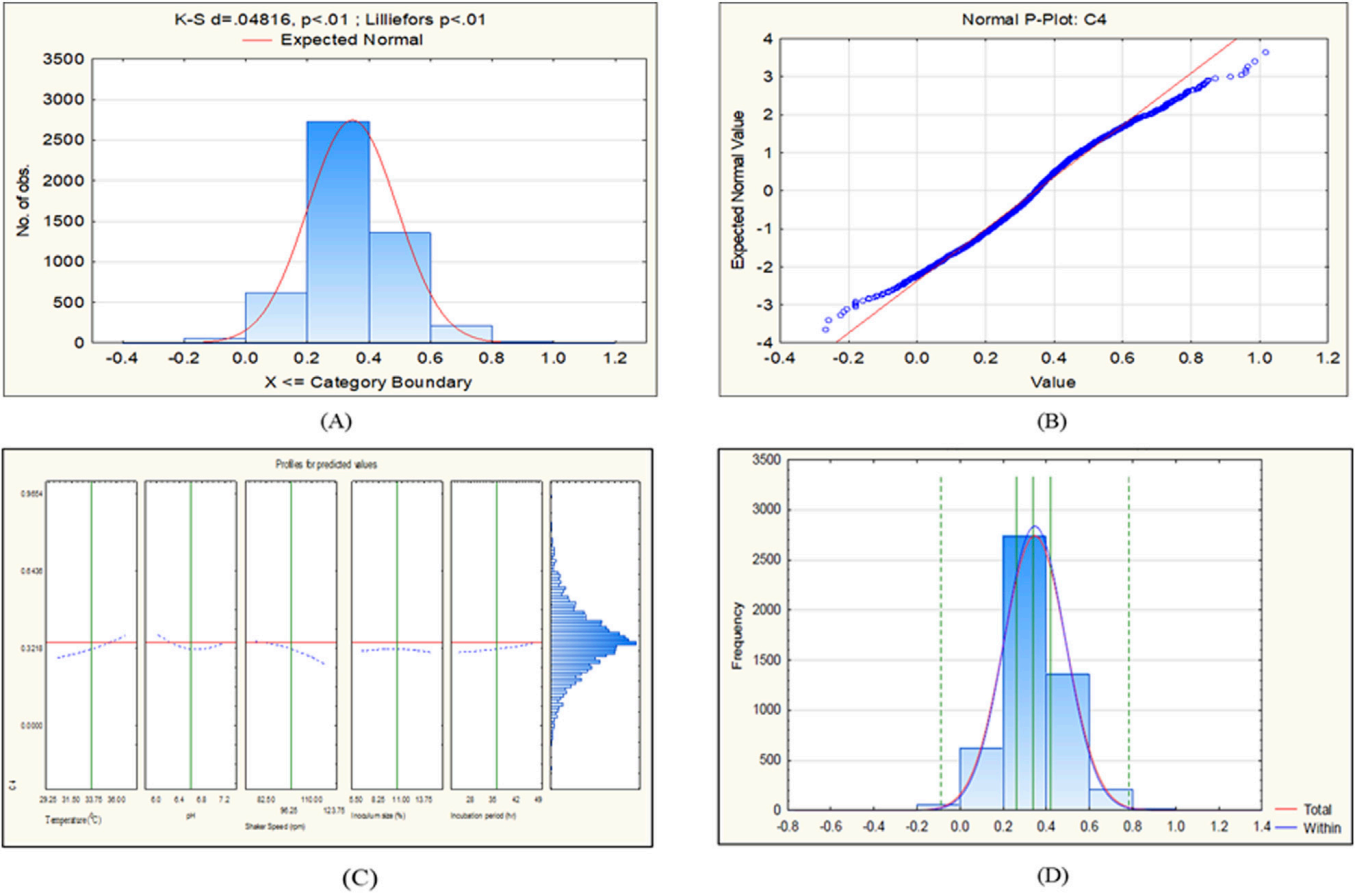
CCD-JT analysis for C4. **(A)** p-value for normal distribution based on Lilliefors test, **(B)** Fitted normal distribution profile for predicted and observed values, **(C)** Predictive optimization of growth conditions, **(D)** Capability test analysis.

**FIGURE 7:**
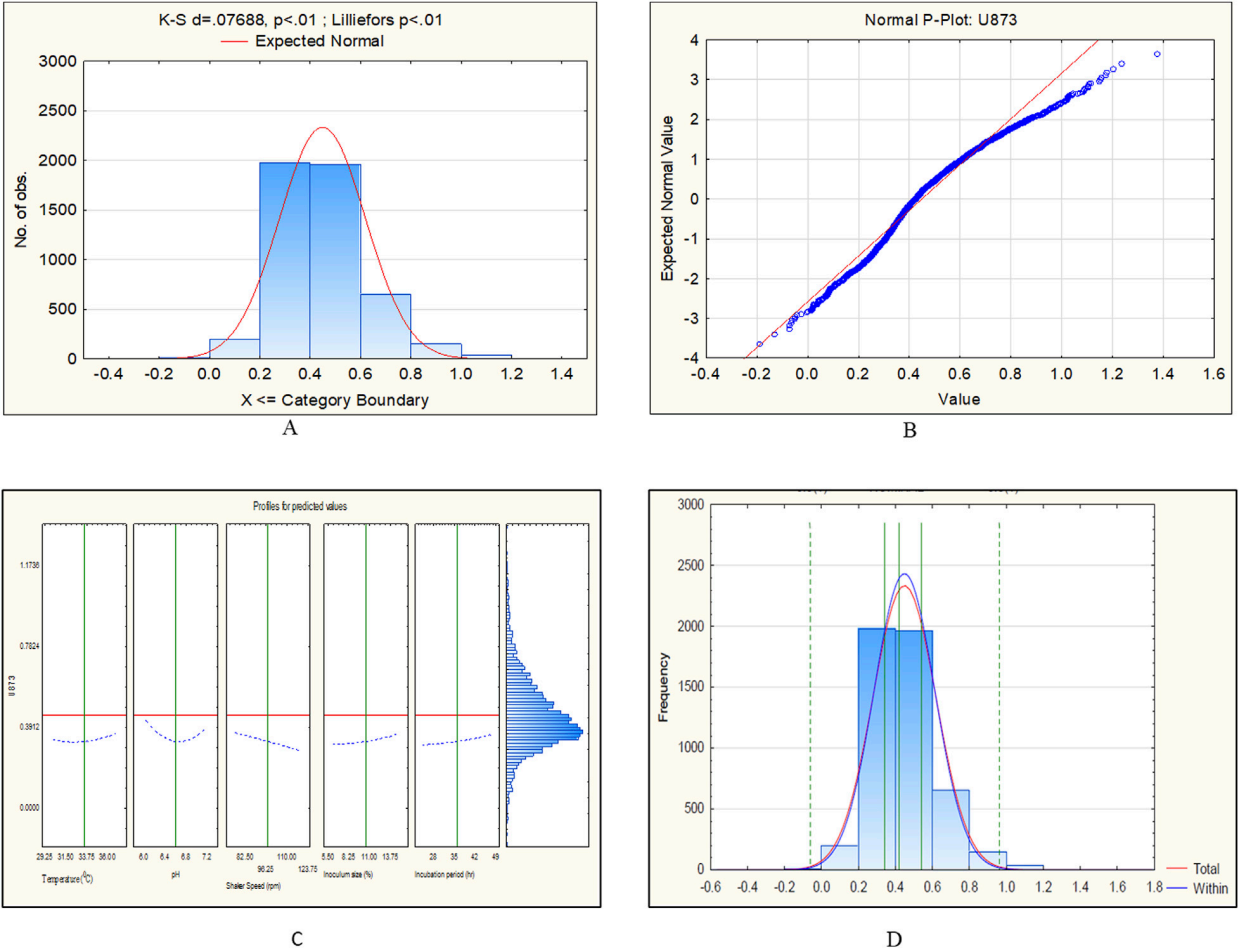
CCD-JT analysis for *Candida tropicalis* U873. **(A)** p-value for normal distribution based on Lilliefors test, **(B)** Fitted normal distribution profile for predicted and observed values, **(C)** Predictive optimization of growth conditions, **(D)** Capability test analysis.

**FIGURE 8:**
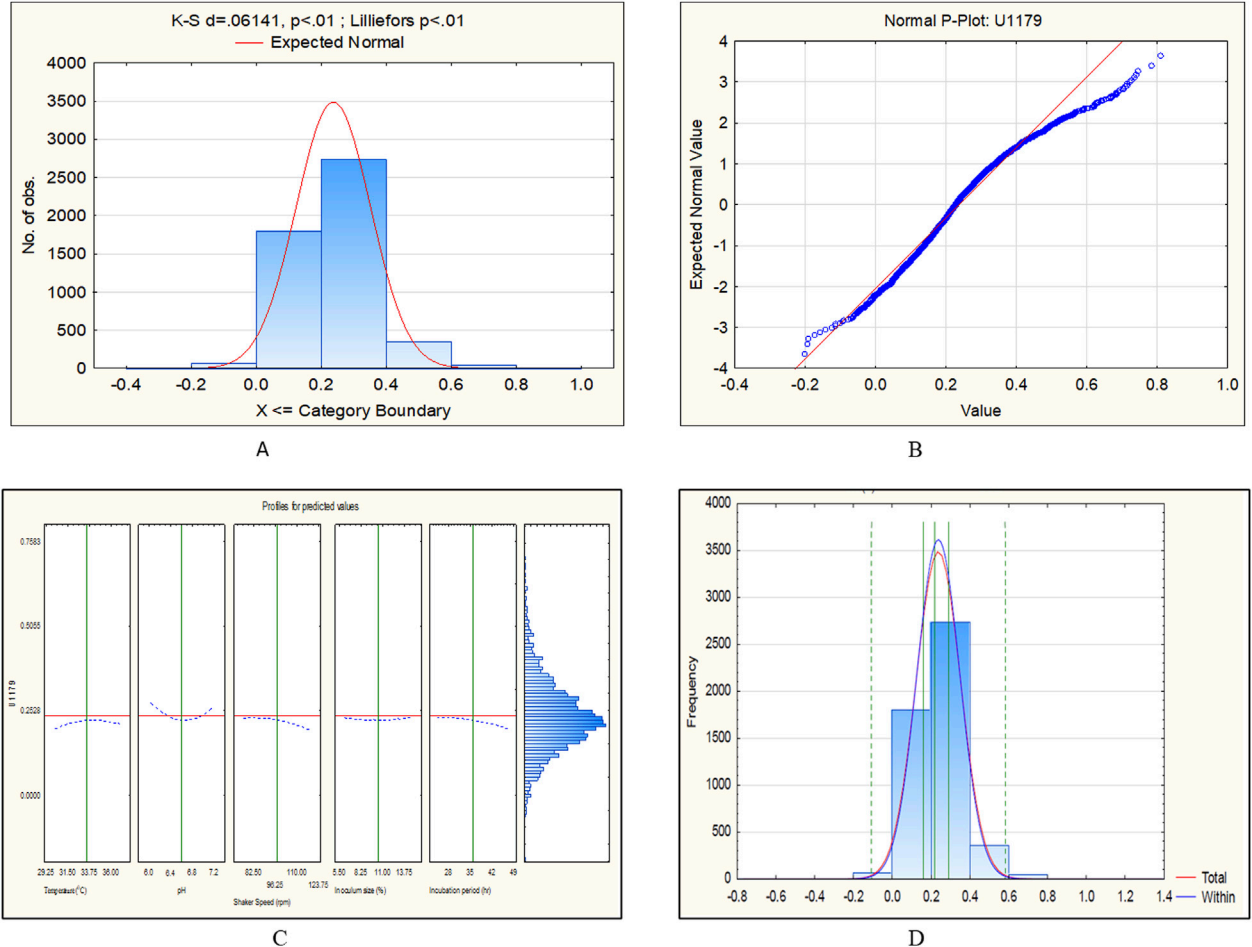
CCD-JT analysis for U1179. **(A)** p-value for normal distribution based on Lilliefors test, **(B)** Fitted normal distribution profile for predicted and observed values, **(C)** Predictive optimization of growth conditions, **(D)** Capability test analysis.

**FIGURE 9:**
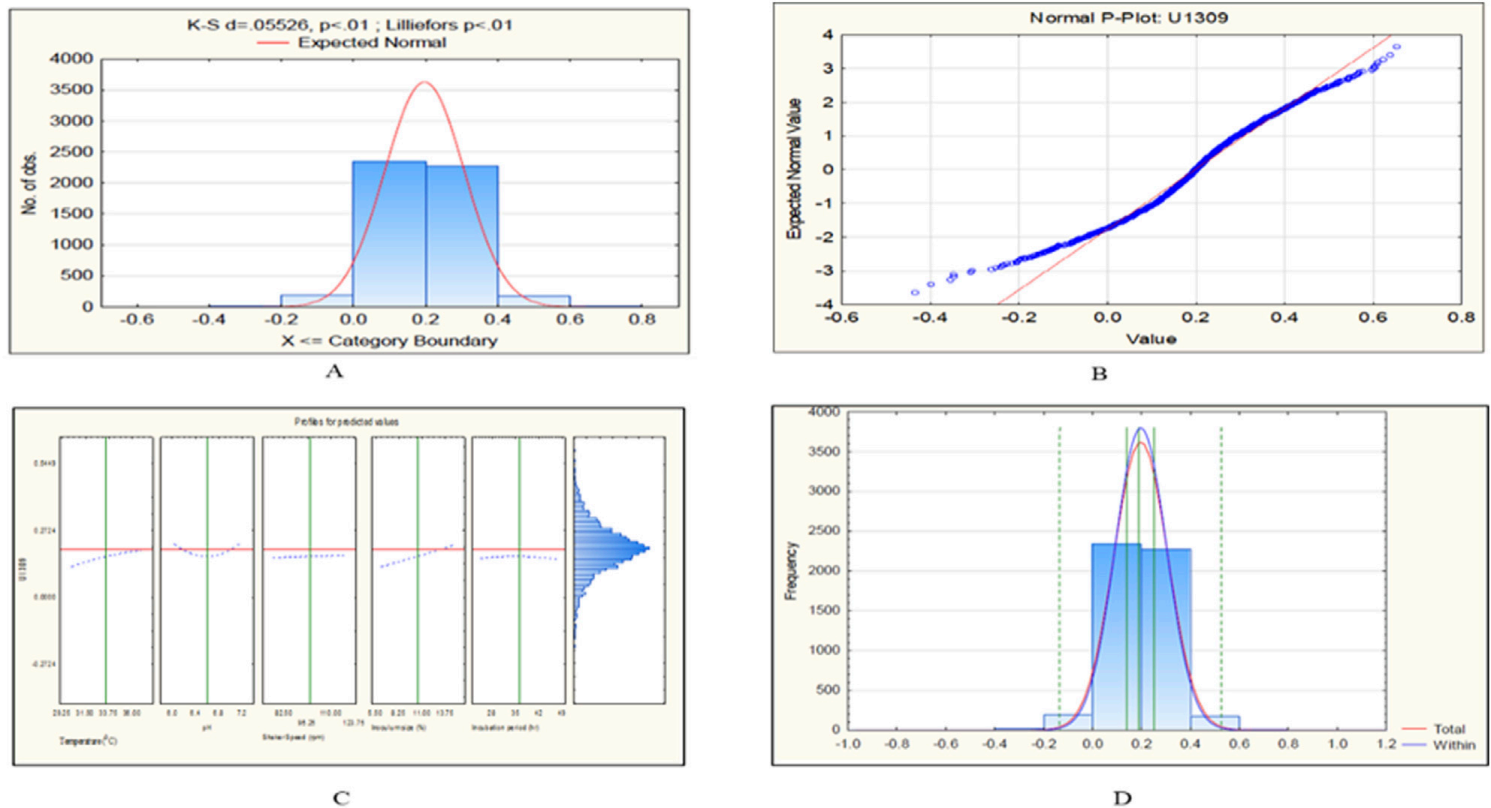
CCD-JT analysis for U1309. **(A)** p-value for normal distribution based on Lilliefors test, **(B)** Fitted normal distribution profile for predicted and observed values, **(C)** Predictive optimization of growth conditions, **(D)** Capability test analysis.

**FIGURE 10:**
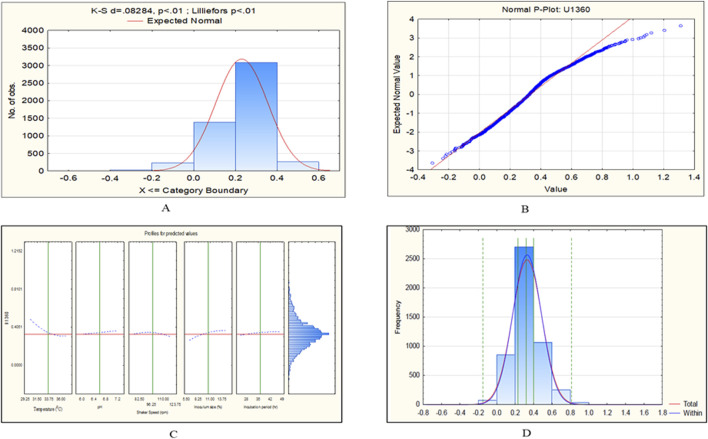
CCD-JT analysis for U1360. **(A)** p-value for normal distribution based on Lilliefors test, **(B)** Fitted normal distribution profile for predicted and observed values, **(C)** Predictive optimization of growth conditions, **(D)** Capability test analysis.

The CCD-JT dataset for the culture MTCC-184 is shown in Figure 5. The CCD–JT analysis has exhibited a p-value of 0.062 for the Lilliefors (K-S d) test (Table 4; Figure 5A). After confirming the reliability of the transformed or untransformed datasets by the Lilliefors test, the CCD-JT predictive model profiler has provided the optimum conditions as shown in (Figure 5B). The capability test analysis for the MTCC-184 transformed datasets predicted a target CV assay value of 0.34, and the observed CV assay value was 0.312 with an SD of ± 0.026 (Table 4; Figures 5C,D). The optimal yield of biofilm by MTCC-184 culture was also calculated using a second-order polynomial regression equation (Equation 2). The CCD–JT analysis datasets for the other cultures C4, U873, U1179, U1309 and U1360 are shown in Figures 6–10 with their respective regression equations (Equations 3–8).

#### 3.3.2 Second-order polynomial regression equation for *Candida tropicalis* cultures based on CCD model profiler (Johnson Transformation)

$$\begin{array}{ll} Y_{\text{MTCC}-184}= & 2.81798+0.260443x_{1}-0.00293932x_{1}^{2}-1.92068x_{2}\\ & +0.127759x_{2}^{2}+0.00767477x_{5}-2.06726e-005x_{5}^{2}\\ & +0.129401x_{4}+0.000330939x_{4}^{2}-0.083614x_{3}\\ & +0.000265288x_{3}^{2}-0.0029861x_{1}x_{2}\\ & -0.000523017x_{1}x_{5}-0.00146693x_{1}x_{4}\\ & +0.000547126x_{1}x_{3}+0.00219289x_{2}x_{5}\\ & -0.00829475x_{2}x_{4}+0.0051302x_{2}x_{3}\\ & -0.00037181x_{5}x_{4}+9.39044e-005x_{5}x_{3}\\ & +0.000312112x_{4}x_{3} \end{array}$$ (3)

$$\begin{array}{ll} Y_{C4}= & 8.40029-0.0698555x_{1}+0.000901362\;x_{1}^{2}-1.68045\;x_{2}\\ & +0.127662x_{2}^{2}-0.0179245x_{5}-3.99176e-005x_{5}^{2}\\ & +0.250028x_{4}-0.000664606x_{4}^{2}--0.102463x_{3}+4.19565e\\ & -005x_{3}^{2}-0.00501985x_{1}x_{2}+0.000544974x_{1}x_{5}\\ & -0.00269312x_{1}x_{4}+0.000867064x_{1}x_{3}+0.000376542x_{2}x_{5}\\ & -0.0243827x_{2}x_{4}+0.00981484x_{2}x_{3}+7.20154e-005x_{5}x_{4}\\ & +5.23146e-005x_{5}x_{3}+0.000202932x_{4}x_{3} \end{array}$$ (4)

$$\begin{array}{ll} Y_{U873}= & 11.521-0.0501711x_{1}+{0.00207936x}_{1}^{2}-3.07655x_{2}\\ & +0.253742x_{2}^{2}-{0.0143463x}_{5}-{5.8986e-006x}_{5}^{2}\\ & +0.138765x_{4}-{0.000632374x}_{4}^{2}-0.00926219x_{3}\\ & +{6.66473e-005x}_{3}^{2}-{0.00525786x}_{1}x_{2}+{0.000469313x}_{1}x_{5}\\ & -0.00347619x_{1}x_{4}+0.000897818x_{1}x_{3}+0.00252778x_{2}x_{5}\\ & -0.015679x_{2}x_{4}+{0.00589698x}_{2}x_{3}+{0.000700412x}_{5}x_{4}\\ & +0.000142438x_{5}x_{3}+0.000148148x_{4}x_{3} \end{array}$$ (5)

$$\begin{array}{ll} Y_{U1179}= & 4.78279+0.218668\;x_{1}-0.00169103x_{1}^{2}-2.09245x_{2}\\ & +0.137828x_{2}^{2}-0.00443767x_{5}-2.74216e-005x_{5}^{2}\\ & +0.0212798x_{4}+0.000398837x_{4}^{2}-0.0630238x_{3}\\ & -6.45732e-005x_{3}^{2}-0.00997024x_{1}x_{2}\\ & -0.000182275x_{1}x_{5\;}-0.00346958x_{1}x_{4}\\ & +0.000463789x_{1}x_{3}+0.00189352x_{2}x_{5}+0.00972991x_{2}x_{4}\\ & +0.00852722x_{2}x_{3}+0.000335186x_{5}x_{4}-2.72375e\\ & -005x_{5}x_{3}-0.000282021x_{4}x_{3} \end{array}$$ (6)

$$\begin{array}{ll} Y_{U1309}= & 5.39136+0.108124x_{1}-0.000921774x_{1}^{2}-2.36542x_{2}\\ & +0.154861x_{2}^{2}-0.0061382x_{5}-2.8807e-006x_{5}^{2}\\ & +0.0509157x_{4}+5.34949e\;005x_{4}^{2}+0.0314841x_{3}\\ & -8.56492e-005x_{3}^{2}+0.00456348x_{1}x_{2}+4.28579e\\ & -005x_{1}x_{5}-0.00333597x_{1}x_{4}-0.000992064x_{1}x_{3}\\ & +0.000435181x_{2}x_{5}+0.0107561x_{2}x_{4}+0.000439818x_{2}x_{3}\\ & +3.37463e-005x_{5}x_{4}+6.2809e-005x_{5}x_{3}\\ & -0.000122684x_{4}x_{3} \end{array}$$ (7)

$$\begin{array}{ll} Y_{U1360}= & 1.51067+0.14942x_{1}-0.00134014x_{1}^{2}-1.04481x_{2}\\ & +0.0723382x_{2}^{2}+0.00544585x_{5}-8.42798e-005x_{5}^{2}\\ & +0.0177725x_{4}-0.000841568x_{4}^{2}-0.0486094x_{3}\\ & -0.000139468x_{3}^{2}-0.00609125x_{1}x_{2}-9.57667e005x_{1}x_{5\;}\\ & -0.00207937x_{1}x_{4}+0.000602182x_{1}x_{3}\\ & +0.000580241x_{2}x_{5}+0.0106019x_{2}x_{4}+0.00451389x_{2}x_{3}\\ & +0.000401236x_{5}x_{4}+0.000159259x_{5}x_{3}\\ & -0.000928238x_{4}x_{3} \end{array}$$ (8)

Note: Y = CV value for biofilm formation, $x_{1}$ = Temperature, $x_{2}$ = pH, $x_{3}$ = Incubation period, $x_{4}$ = Inoculum size, $x_{5}$ = Shaker speed

### 3.4 Quantification of *Candida tropicalis* biofilm

With the optimized growth conditions given by the CCD model and CCD-JT, further quantification of *C. tropicalis* biofilm on silicone elastomer material was carried out by different assays. Biofilm formation was measured based on CV assay, cell viability by MTT assay, and cell mass by calcofluor assay. Cell mass was also calculated by wet and dry weight measurements.

**FIGURE 11:**
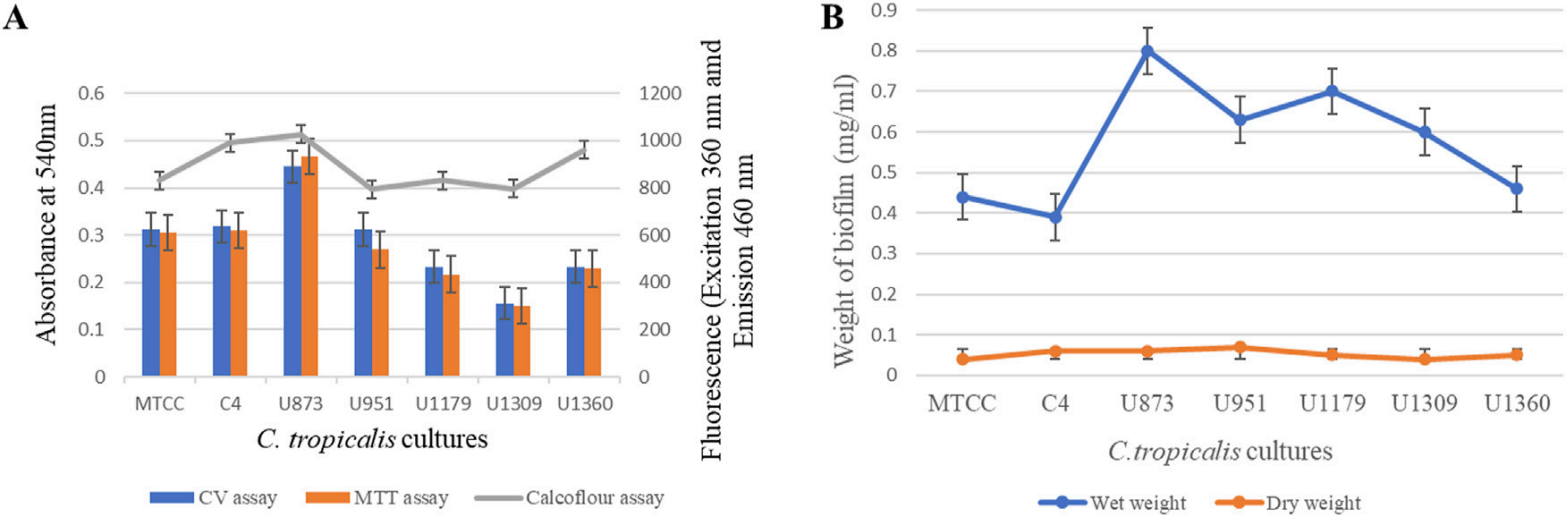
Quantification of *Candida tropicalis* biofilm on silicone elastomer material. **(A)** CV, MTT and Calcofluor assays; **(B)** Wet weight and Dry weight measurements.

Based on the CV assay, *C. tropicalis* C4, U873 and MTCC-184 cultures were categorized as high biofilm formers, and U951, U1360 as intermediate biofilm formers and U1179, U1309 as low biofilm formers. This categorization of high, intermediate, and low biofilm formers also correlates with the biofilm tube test. A direct correlation was observed between CV and MTT assays for all the *C. tropicalis* cultures, as observed in Supplementary Table S1 and Figure 11, indicating that a linear relationship is observed between biofilm formation and cell viability. The quantification of cell mass based on the calcofluor assay and wet and dry weight measurements showed that *C. tropicalis* U873 and C4 not only have high viability and biofilm formation, but also produce high cell mass. However, MTCC-184 has moved to the intermediate category with the calcofluor assay and the low category in case of the wet and dry weight measurements. As per the Calcofluor assay, *C. tropicalis* U1360, an intermediate biofilm former, had high cell mass but gravimetric measurements indicate otherwise.

The ranking order for the biofilm formation of *C. tropicalis* cultures based on the CV assay is: U873 > C4>MTCC-184 > U951 > U1360 > U1179 > U1309.

The ranking order for the cell viability of *C. tropicalis* cultures based on the MTT assay is: U873 > C4>MTCC-184 > U951 > U1360 > U1179 > U1309.

The ranking order for cell mass of *C. tropicalis* cultures with Calcofluor assay is:

U873 > C4>U1360>MTCC-184 > U1179 > U1309 > U951.

The ranking order for the wet weight-based cell mass of the *C. tropicalis* cultures is: U873 > U1179 > U951 > U1309 > U1360 > MTCC-184 > C4.

The ranking order for the dry weight associated cell mass of the *C. tropicalis* cultures is: U951 > U873 > C4>U1179 > U1360 > MTCC-184 > U1309.

### 3.5 Discussion

The formation of biofilm by *C. albicans* and NAC species has been linked to the pathogenicity of persistent and recurring infections (JS and AP, 2011; Soll and Daniels, 2016; Pereira et al., 2021). Amongst the NAC, *C. tropicalis* is the most commonly encountered species in several Asian nations (Sharma and Chakrabarti, 2023). Most hospital-acquired infections and infections linked to medical devices are caused by *C. tropicalis*, which forms biofilm by attaching itself to both biotic and abiotic surfaces, more so in immunocompromised individuals. The extensive use of a wide variety of medical implant devices has almost paralleled the increase of *Candida* infections in the population with impaired host defences (Malinovská et al., 2023). Studies indicate that the presence of an invasive medical device is the key source for developing invasive candidemia (Sherry, 2014; Soulountsi et al., 2021). Biofilms produced by the *C. tropicalis* organism form a shield to protect from various environmental stresses, inducing virulence and pathogenicity. The probable reason behind this would be the ability of the organism to undergo switching mechanisms when the living environment is harsh and continue to adapt easily and survive (Soll and Daniels, 2016; Luo et al., 2021) and/or undergo gene upregulation and metabolic heterogeneity (Fanning and Mitchell, 2012; Moralez et al., 2020). According to recent studies, biofilm growth is significantly influenced by coordinated metabolic stress regulation mechanisms (Rather et al., 2021). Biofilms’ strong resistance to antimicrobial drugs is one of their most significant characteristics (Zuza-Alves et al., 2017).

Clinical isolates of *C. tropicalis* sourced from blood, urine, and sputum of patients with invasive candidiasis are associated with biofilm-related infections, such as systemic, urinary tract infections, and respiratory conditions were used for the study. The correlation between cell viability and biofilm formation in *C. tropicalis* is a significant aspect of its pathogenicity. As per the microbial characteristics, host interactions, and clinical outcomes, C4 and U873 were found to be highly virulent biofilm formers. U873 was also found to be resistant to flucytosine as per VITEK AST. U951 and U1360 were found to be virulent intermediate biofilm formers. U951 was also found to be resistant to flucytosine as per VITEK AST. U1179 and U1360 were found to be moderately virulent, low biofilm formers. The descending order of biofilm formation and virulence of *C. tropicalis* clinical isolates is as follows: U873 > C4> U951 > U1360 > U1179 > U1309. For biofilm formation studies, TSB medium is used, as it provides essential nutrients (amino acids, vitamins, carbohydrates) from tryptone, soy peptone, and dextrose, creating favorable conditions for *C. tropicalis* growth and initial biofilm adhesion. Therefore, this medium was selected for this study as it supported maximum biofilm formation in *C. tropicalis* compared to YEPD and SDA. In a previous study with polymicrobial biofilm (*C. albicans* M-207 and *Escherichia coli* ATCC 39936), we observed that polymicrobial biofilms grow well in TSB medium (Ashrit et al., 2022), which also makes it suitable for cross-kingdom biofilm studies.

Quantitative and qualitative analysis of parameters one at a time using the traditional OVAT studies is often expensive and tedious, with statistically significant random and systemic errors. Most importantly, in OVAT studies, the interaction effects of the variable under consideration are most often neglected. The present study plays a significant role in understanding the growth conditions and their interactions that aid in the formation of biofilms produced by *C. tropicalis*. The growth parameters considered for this study include temperature, pH, shaker speed, inoculum size, and incubation time. Each parameter is tested in five subsets. We have used RSM, employing CCD to design our experiments. The experimental trials were designed using the CCD-based RSM model, which considers the factorial components of the axial and centre points. The resultant output will be significant in terms of resolution since factorial portions of variables (interaction effects) are considered and included in the design of experiments. In other CCD models, such as the Box and Draper and Plackett-Burman models, it is frequently challenging to attain a similar resolution of independent variables. We tried with Box-Cox transformation, however, it showed the lambda value of −2 (not equal to 1) that necessitates for further data transformation for the current study. Therefore, we have used the CCD model profiler tool (Johnson Transformation) to overcome the non-normality. The CCD model profiler tool of the Statistica software has a unique interface that helps to ‘Edit correlation’ and ‘Fit best distribution’. This tool enabled us to find the best transformation mode with the best fit, which has transformed all the predictor distributions at the defined five level. This tool enabled us to get a normal distribution of the skewed datasets obtained from the CCD model. Later, a regression equation that produces the best outcome is generated.

For the U951 isolate, the optimized conditions as per the CCD-based RSM model are: Temperature of 32.7°C, pH- 7.14, Shaker speed- 110 rpm, Inoculum size – 14% and Incubation time- 38.5 h. In the present investigation, we observed that among the selected physico-chemical factors, 1L and 5L, i.e., temperature and incubation period, were found to be significant for U951. This outcome indicates that mesophilic temperature and incubation period for interaction are critical as compared to pH and shaker speed. However, all of the *C. tropicalis* isolates are able to grow in the unified optimal conditions given by the Johnson transformation (Temperature of 35°C, pH- 6.6, Shaker speed – 38 rpm, Inoculum size- 7%, Incubation time- 45 h). However, there could be a strain-wise difference with reference to specific parameters impacting biofilm formation. *C. tropicalis* clinical isolates demonstrate optimal biofilm formation at a temperature of 30°C–37°C, mirroring human body conditions and supporting growth, adhesion, and virulence. Lower temperatures reduce metabolic activity and biofilm formation, while higher temperatures induce stress, impairing viability and biofilm structure (Casagrande Pierantoni et al., 2021). A media pH of 6-7 was optimal for *C*. *tropicalis* growth, supporting balanced enzyme activity and cell metabolism. At lower pH levels, acidic stress can inhibit growth and biofilm formation, while higher pH may disrupt membrane integrity and metabolic functions, leading to reduced viability (Tseng et al., 2020; Ashrit et al., 2022). An optimum shaker speed of 40–80 rpm supports ideal oxygen transfer and nutrient distribution for *C. tropicalis* growth. Higher speeds may cause shear stress, disrupting cell adhesion and biofilm integrity, while lower speeds can reduce oxygen availability, leading to slower growth and weaker biofilm formation (Sadanandan et al., 2022; Santos et al., 2016). An Inoculum density of 7% inoculum size was optimal for *C*. *tropicalis* biofilm formation, likely due to a balance between sufficient cell density and favorable environmental conditions. Higher inoculum levels may cause nutrient depletion and waste accumulation, disrupting biofilm structure, while lower levels support robust development through reduced competition and effective quorum sensing (Sadanandan et al., 2022; Tseng et al., 2020; Li and Zhao, 2020). A 24–48 h incubation optimally supports *C. tropicalis* biofilm maturation. Shorter durations may yield immature, weak biofilms, while longer durations can cause nutrient depletion and biofilm degradation, reducing structural integrity and cell viability (Malinovská et al., 2023).

Based on CCD optimization, validating the model using ANOVA is important. The ANOVA analysis ensures the information generated is unbiased and further confirms the significance of the variable observed during optimization. ANOVA-based statistical analysis typically analyzes the input data based on the p-value and F-value. The p-values of the independent variables are as follows: ***p < 0.001 (very highly significant), **p < 0.01 (highly significant), and *p < 0.05 (significant). Although our CCD-integrated ANOVA analysis did not demonstrate strong statistical significance, it still meets the minimum threshold for significance (p < 0.05). The outcome of ANOVA is further substantiated by the Pareto chart of standardized effect as shown in (Figure 1; Table 2) for the U951 isolate, where the interaction of 1L by 5L has reached the standardized effect of p = 0.049 rounded off to 0.5 by default by the tool. In several cases or studies that involve biological or physiology-related experiments, CCD outcomes exhibit inconsistent results. This evidence is well documented by an official report published by the Aviation Research Laboratory, UIC [https://apps.dtic.mil/sti/tr/pdf/AD0748277.pdf] and other related recent publications. Though CCD is a robust and flexible design, in microbial factors (commensals in humans in this case), CCD may give inconsistent or variable results. This problem we have tried to troubleshoot by using a data transformation tool (Breig and Luti, 2021; Kean et al., 2018).

In general, RSM studies lead to dynamic results as they are related to non-linear microbial culture studies. The microbial cultivation profile of *Candida* spp. typically follows a non-linear system (Manns et al., 2014; Sorour et al., 2023). For RSM studies, mean and variance are highly critical for successful process optimization. The culture system possessing non-linear trends exhibits a high degree of heterogeneity and does not follow normal distribution traits. Thus, there is a need for stabilization of the mean and variance, which is feasible through stabilized data transformation. The data transformation process effectively fits the variance of distribution and heterogeneity to a mean value, and the obtained processing variable can provide optimal conditions for process optimization (Liu, 2009; Zhang et al., 2023). In the present investigation, we have used mathematical transformation for the dependent variable(s) that follow normal distribution traits during routine CCD analysis. The variance of distribution and heterogeneity among independent variables can be brought to a mean by data transformation studies. In this study, we observed that the datasets of *C. tropicalis* cultures such as MTCC-184, C4, U873, U1179, U1309, and U1360 after CCD-JT exhibited p value of α ≥ 0.10. We have observed that CCD-JT gave a unified optimal growth condition but different predicted values. This is probably due to the principles of the statistical transformation process, where variance and heterogeneity are pooled into a mean (Liu, 2009; Cai and Xu, 2024).

A study by (Mancera et al., 2015; Tseng et al., 2020) on *C. tropicalis* biofilms was unable to establish stable biofilms on the bottom of polystyrene plates. However, on a silicone-based substrate, they were able to strongly promote biofilm formation. In this study, we have also observed that *C. tropicalis* cultures were able to grow on silicone elastomer material with the optimal growth conditions given by the CCD model and CCD-JT. A detailed comparison of the ideal culture conditions of *Candida species* based on biofilm formation, growth and cell viability on different types of polymeric material is shown in (Table 5). Silicone elastomer is often chosen as a substrate for biofilm studies due to its unique material properties, such as surface hydrophobicity and elasticity that can influence microbial adhesion and biofilm formation (Tseng et al., 2020). This material tends to have more biofilm formation due to its ability to absorb strain energy, allowing the biofilm to withstand transient stress events like fluid flow by conforming to the surface deformation, creating a more stable environment for the colonization of *Candida* species (Zare et al., 2021). *C. tropicalis* cell surface hydrophobicity is the tendency of the cells to interact with hydrophobic surfaces, such as host cells/tissues and medical devices. Higher hydrophobicity generally leads to increased adhesion, enhanced biofilm formation, and greater virulence. The comparison of the hydrophobicity of *C. tropicalis* with other *Candida* species is shown in (Table 6).

**TABLE 5:** Optimum growth conditions of *Candida* species on polymeric materials.

*Candida* species	Material	Temperature (°C)	pH	Inoculum size	Shaker speed (rpm)	Incubation time (h)	Reference
*C. tropicalis*	Polystyrene microtiter plate	37	7	1 × 10^7^ CFU/mL	Static	24, 48, 72, and 96	Atiencia-Carrera et al. (2022)
*C. albicans*							
*C. tropicalis*	Polystyrene microtiter plate	37	6.9	OD_600_ of 0.1	100	24–48	Tan et al. (2016)
*C. krusei*							
*C*.*parapsilosis*							
*C. tropicalis*	Silicone-based platform	30	7	OD_600_ of 0.5 1 × 10^7^ cells/square	100	48	Tseng et al. (2020)
*C. tropicalis*	Polypropylene	37	7	1 × 10^6^ cells/mL	Static	24–48	Vera-González and Shukla (2020)
*C. albicans*							
*C. glabrata*							
*C. krusei*							
*C. albicans*	Polystyrene microtiter plate	25–35	6.5	1 × 10^6^ cells/mL (Pre-inoculum stock density) 7%, 10%, 13% (Inoculum percentage)	40–80	42–77	Sadanandan et al. (2022)
*C. albicans*	Polystyrene microtiter plate	37	6–8	1 × 10^6^ cells/mL	100	24	Ashrit et al. (2022)
*C. albicans*	Polypropylene	37	6.3–6.7	1 × 10^6^ cfu/mL	100	72	Slettengren et al. (2020)
*C. albicans*	Titanium Alloy (Ti-6Al-4 V)	37	5.6	OD_600_ of 0.08–0.1 1 × 10^6^ cfu/mL	100	48	Morais et al. (2025)
*C. glabarata*	polystyrene microtiter plates	30	5.5–8.5	1 × 10^6^ cells/mL (cell density)	60	24	Sadanandan et al. (2021)

**TABLE 6:** Comparative hydrophobicity, biofilm formation, and adherence of *Candida* species on polymeric materials.

*Candida* species	Relative cell surface hydrophobicity	Biofilm-forming ability	Adherence to polymeric materials	References
*C. tropicalis*	High	Strong	High (polyurethane, PVC, silicone elastomer)	Bezerra et al., 2020; Tseng et al., 2020
*C. auris*	Moderate to High	Strong	High (latex, nitrile surfaces)	Malinovská et al. (2023)
*C*. *albicans*	Moderate to High	Strong	High (silicone, polystyrene, PVC, dentin)	Sadanandan et al. (2022)
*C. parapsilosis*	Moderate	Moderate to Strong	High (Teflon, plastic surfaces	Dabiri et al. (2018)
*C. glabrata*	Low to Moderate	Weak to Moderate	Moderate (polystyrene, PVC)	Timmermans et al. (2018)
*C. krusei (Pichia kudriavzevii)*	Variable (Low to Moderate)	Weak to Moderate	Moderate (polyethylene, PVC)	Malinovská et al. (2023)

Using one assay alone does not provide a comprehensive dataset to understand the complexity of biofilm. Hence, selecting assays that can complement one another is crucial. The results from each of these assays provide a thorough analysis of specific cultures under consideration. Therefore, quantification of *C. tropicalis* biofilm on silicone elastomer material was validated by performing different assays as per the optimized conditions.

Based on the results of MTT and CV assay, direct correlation was observed between biofilm formation, and cell viability for all the *C. tropicalis* cultures. The complicated structure of the *C. tropicalis* biofilm, which comprises of a mixture of viable, dormant, and dead cells in addition to the extracellular matrix, can be attributed to the direct correlation observed between biofilm formation and cell viability (Araújo et al., 2017). Whereas viability shows the cells that are actively metabolising inside the biofilm, which may or may not comprise most of the biofilm but biomass reflects the overall structure and cell mass of the biofilm (Tseng et al., 2020).

However, based on cell mass there is a slight change in the ranking order of *C. tropicalis* cultures which can be attributed to a combination of factors such as genetic diversity of the cultures, variations in biofilm production of cultures and the composition of the yeast cell wall especially chitin content (de Souza et al., 2022). The variation in the ranking order might also be due to varying levels of EPS production, water retention capabilities, biofilm density, growth conditions, and metabolic behavior of the *C. tropicalis* cultures (Tseng et al., 2020). According to (Silva-Dias et al., 2015), species-specific variations are seen with respect to the development of biofilms. We have observed strain specific variation in biofilm formation. Our study has revealed that some of the invasive *C. tropicalis* strains exhibit high biofilm, metabolic activity and cell mass offsetting the general notion that virulence and cell viability are inversely proportional (Sadanandan et al., 2021; Ashrit et al., 2022). These findings further highlight the heterogeneity and the evolving adaptability of this pathogen making it one of the most virulent and fast-growing *Candida* species.

*C. tropicalis* cultures grown on silicone elastomer material in their optimal conditions have a variety of industrial applications such as screening of therapeutics and antimicrobial agents, in the development of biomaterials and coatings, in the pharmaceutical and food industry, wastewater treatment and bioremediation (Almeida et al., 2017; Madian et al., 2019). We can implement the *in vitro* model on silicone elastomer for high-throughput screening of therapeutic agents by integrating one or more of the following–upscaling/miniaturization, automation and facilitating *in situ* or real-time quantitative readouts.

We can convert these results into useful applications by developing biofilm-resistant medical devices, enhancing anti-biofilm treatments (antimicrobial lock strategies, that effectively disrupt biofilms under specific conditions optimized for their growth (Sadanandan et al., 2025). In the NCBI SRA portal, apart from the whole genome sequence of the four *C. tropicalis* isolates submitted by us we only find *C. tropicalis* MYA 3404 in the clinical isolate category, therefore, there is a need for more sequencing and annotation studies. There is also scope for research in the area of interaction of *C. tropicalis* cells and biofilm matrix with polymeric materials used in medical devices.

### 3.6 Conclusion

In this study, RSM was used for the optimization of *C. tropicalis* growth conditions on silicone elastomer material. Temperature, media pH, shaker speed, inoculum size, and incubation time were the chosen growth conditions. RSM is a statistical and mathematical technique used to develop models, evaluating the impact of multiple independent variables, and it determines the ideal value for each variable. We observed that of the seven strains, only *C. tropicalis* U951 was statistically significant based on Pareto chart analysis and ANOVA, and the remaining six strains of *C. tropicalis,* namely, MTCC-184, C4, U873, U1179, U1309 and U1360, were found to be not statistically significant on silicone elastomer material. Thus, we adopted the CCD integrated model profiler, i.e., the Johnson transformation method, which enables us to transform our datasets to follow a normal distribution and to obtain statistically reliable optimized growth conditions for the culturing of *C. tropicalis*. With the optimized conditions, the results were further validated by the quantification of biofilm using MTT assay, CV assay, Calcofluor white assay, Wet weight, and dry weight measurements. A crucial finding not reported earlier in C*. tropicalis* is the direct correlation between biofilm formation and cell viability with variations in the cell mass, paving the way for further research into the complex world of biofilms. Optimizing growth conditions using statistical tools like JT enabled us to establish more precise and reproducible biofilm models that closely mimic clinical settings. Therefore, this will help to understand biofilm formation dynamics and test drug efficacy under optimal conditions, thereby diminishing the scope for drug resistance and also aiding in exploring novel therapeutic strategies.

## Data Availability

The original contributions presented in the study are included in the article/Supplementary Material, further inquiries can be directed to the corresponding author.

## Abbreviations

RSM, Response surface methodology; CCD, Central composite design; JT, Johnson Transformation; CCD-JT, Central composite design integrated Johnson Transformation; OVAT, One Variable at a time; TSB, Tryptic Soy Broth; CV, crystal violet; MTT, 3- (4, 5-dimethylthiazol-2-yl)-2,5-diphenyltetrazolium bromide; K-Sd, Kolmogorov–Smirnov test.
