# Supplementary material for: Optimization of Candida tropicalis growth conditions on silicone elastomer material by response surface methodology
Source: Front Bioeng Biotechnol. 2025 Jul 17;13:1572694. doi: 10.3389/fbioe.2025.1572694 (PMC12311636; doi:10.3389/fbioe.2025.1572694)
Supplement: Supplementary file 1 [file DataSheet1.docx]

Supplementary Material

##
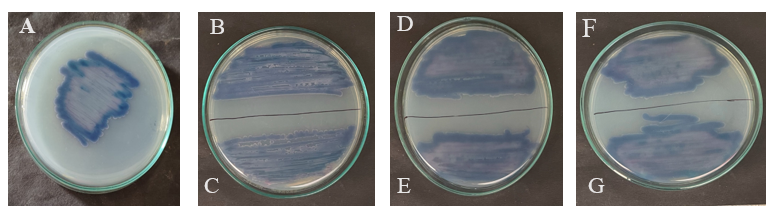
Supplementary Figures

**Supplementary Figure 1.** Identification of *C. tropicalis* isolates on HiCrome™agar. (A) MTCC, (B) C4, (C) U873, (D)U951, (E) U1179, (F) U1309, and (G) U1360.


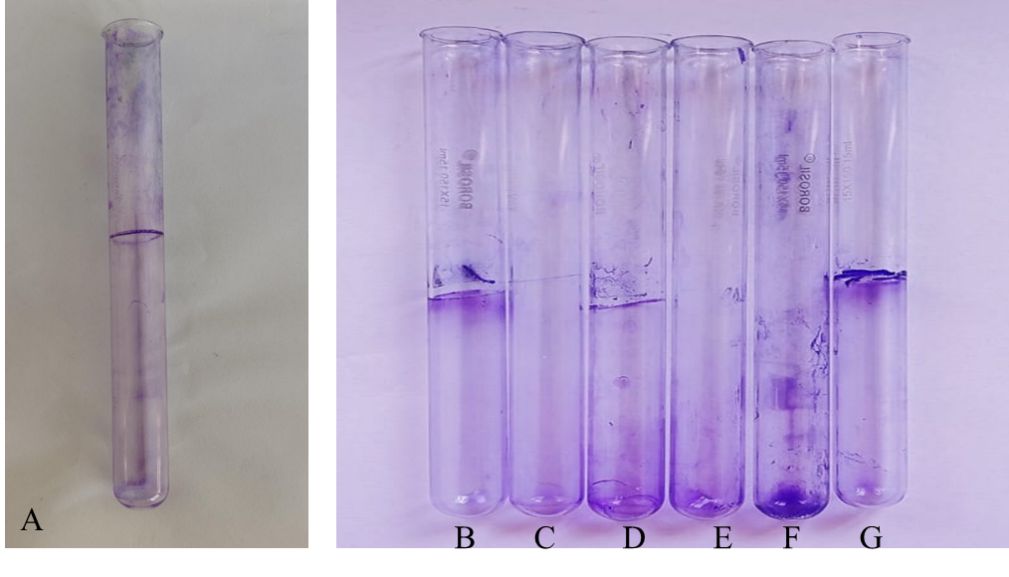


**Supplementary Figure 2.** Biofilm tube test of *C. tropicalis* isolates stained with crystal violet. (A) MTCC, (B) C4, (C) U873, (D)U951, (E) U1179, (F) U1309, and (G) U1360.


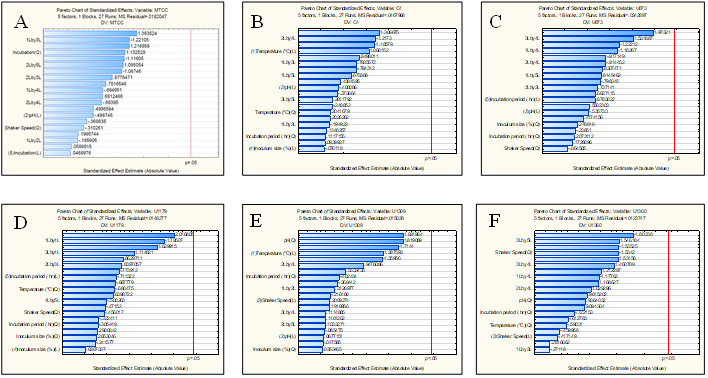


**Supplementary Figure 3.** Pareto chart showing significant independent factor(s) at linear (L) and quadratic (Q) forms for *C. tropicalis* cultures on silicone elastomer material. (A) MTCC-184 (B) C4 (C) U873 (D) U951 (E) U1179 (F) U1309 (G) U1360.

**Supplementary Figure 4.** Growth OD of *C. tropicalis* clinical isolates with different inoculum size percentages

*7% Inoculum size as per CCD- JT

* 14% Inoculum size for U951 isolate via CCD based RSM

**Supplementary Figure 5.** Cell count of *C. tropicalis* clinical isolates with different inoculum size percentages

**1.2 Supplementary Tables**

**Supplementary Table 1.** Response of *C*. *tropicalis* cultures elucidated through CV assay, MTT assay, Calcofluor white binding assay, wet and dry weights of biofilm at predicted conditions given by CCD model and CCD-JT.

| ***C*. *tropicalis* cultures** | **CV assay** | **MTT assay** | **Calcofluor assay** | **Wet weight** | **Dry weight** |
| --- | --- | --- | --- | --- | --- |
| MTCC-184 | 0.312±0.026 | 0.305±0.043 | 832.666 ± 129.515 | 0.44 | 0.04 |
| C4 | 0.318±0.003 | 0.31±0.004 | 992 ±55.973 | 0.39 | 0.06 |
| U873 | 0.445±0.008 | 0.467±0.112 | 1026 ± 126.716 | 0.8 | 0.06 |
| U951 | 0.312±0.026 | 0.269 ± 0.136 | 793.333 ± 62.139 | 0.63 | 0.07 |
| U1179 | 0.233±0.026 | 0.217 ± 0.025 | 831± 388.222 | 0.7 | 0.05 |
| U1309 | 0.156±0.007 | 0.15 ± 0.053 | 796.666 ± 55.752 | 0.6 | 0.04 |
| U1360 | 0.233±0.026 | 0.229 ± 0.017 | 959.666 ± 60.144 | 0.46 | 0.05 |

**Supplementary Table 2. Properties of the silicone elastomer material**

| **Property** | **Test method** | **Value (FPS)** | **Test method** | **Value (Metric)** |
| --- | --- | --- | --- | --- |
| Density | ASTM D297 | 1.20 lb/ft^2^ | DIN 53508 | 1.20 g/cm^3^ |
| Hardness | ASTM D2240 | 60 ± 5 Shore A | DIN 53505 | 60 ± 5 shore A |
| Tensile Strength (Min) | ASTM D412 | 855 PSI | DIN 53504 | 60 kg/cm^2^ |
| Elongation @Break (Min) | ASTM D412 | 250% | DIN 53504 | 250% |
| Abrasion Resistance (Max) | ASTM D5963 | N/A | DIN 53516 | N/A |
| Compression Set (70 ͦ C/ 24hrs/25% Compression) (Max) | ASTM D395 Method B | 30% | DIN 53517 | 30% |
| Tear Resistance (Angular, Min) | ASTM D624 | 140 lbs/inch | DIN 53515 | 25kg/cm |
| **Change in Properties (after heat ageing for 72 hrs/ 100 ͦ C)** | **Test method** | **Value (FPS)** | **Value (Metric)** |  |
| Hardness | ASTM D573 | + 2 pts (Max) | + 2 pts (Max) |  |
| Tensile strength |  | + 5/ -5% | + 5/ -5% |  |
| Elongation @ Break |  | + 5/ -5% | + 5/ -5% |  |
| **Volume Swell (after 72 h/ 70 °C)** | **Test method** | **Value (FPS)** | **Value (Metric)** |  |
| ASTM Oil No.1 | ASTM D471 | Not recommended | Not recommended |  |
| ASTM Oil No. 1 |  | Not recommended | Not recommended |  |
| ASTM Oil No.1 |  | Not recommended | Not recommended |  |
| **Chemical Resistance** | **Test method** | **Value (FPS)** | **Value (Metric)** |  |
| Ozone | ASTM 01149 | Excellent | Excellent |  |
| Dilute Acids & Bases |  | Excellent | Excellent |  |
| Concentrated Acids and Bases |  | Excellent | Excellent |  |
| Oils |  | Excellent | Good |  |
| Solvents |  | Excellent | Fair |  |

**Supplementary Table 3.** Growth OD of *C. tropicalis* clinical isolates with different inoculum size percentages

| ***C. tropicalis* isolates** | 1.50% | 6% | 7%* | 10.50% | 14.00%* | 15% | 19.50% |
| --- | --- | --- | --- | --- | --- | --- | --- |
| MTCC | 0.09±0.007 | 0.12±0.006 | 0.14±0.008 | 0.2±0.009 | 0.25±0.007 | 0.29±0.009 | 0.35±0.019 |
| C4 | 0.08±0.009 | 0.18±0.007 | 0.23±0.013 | 0.28±0.010 | 0.34±0.008 | 0.37±0.007 | 0.45±0.002 |
| U873 | 0.09±0.021 | 0.19±0.013 | 0.22±0.007 | 0.29±0.002 | 0.36±0.007 | 0.38±0.008 | 0.47±0.007 |
| U951 | 0.07±0.008 | 0.09±0.007 | 0.14±0.007 | 0.18±0.013 | 0.25±0.021 | 0.29±0.002 | 0.35±0.009 |
| U1179 | 0.08±0.021 | 0.11±0.007 | 0.15±0.002 | 0.2±0.026 | 0.24±0.004 | 0.27±0.007 | 0.33±0.019 |
| U1309 | 0.07±0.013 | 0.09±0.006 | 0.11±0.002 | 0.12±0.007 | 0.2±0.012 | 0.24±0.008 | 0.3±0.004 |
| U1360 | 0.08±0.008 | 0.13±0.014 | 0.18±0.006 | 0.22±0.010 | 0.26±0.014 | 0.29±0.008 | 0.32±0.007 |

*7% Inoculum size as per CCD- JT

* 14% Inoculum size for U951 isolate via CCD based RSM

**Supplementary Table 4.** Cell count of *C. tropicalis* clinical isolates with different inoculum size percentages

| ***C.tropicalis* isolates** | 1.50% (×10^6^ ) | 6%  (×10^6^ ) | 7% (×10^6^ ) | 10.50% (×10^6^ ) | 14.00% (×10^6^ ) | 15%  (×10^6^ ) | 19.50%  (×10^6^ ) |
| --- | --- | --- | --- | --- | --- | --- | --- |
| MTCC | 4.85±0.035 | 5.2±0.070 | 5.4 ±0.014 | 5.6±0.084 | 5.9±0.070 | 6.35±0.035 | 7.2±0.007 |
| C4 | 4.9±0.070 | 5.4±0.014 | 5.65±0.021 | 5.75±0.035 | 5.85±0.007 | 6±0.014 | 6.27±0.035 |
| U873 | 4.5±0.056 | 6.62±0.021 | 6.6±0.014 | 7±0.070 | 7.5±0.042 | 8.3±0.014 | 9.1±0.007 |
| U951 | 3.55±0.014 | 3.95±0.021 | 4±0.007 | 4.52±0.014 | 4.6±0.014 | 5.2±0.021 | 6.12±0.014 |
| U1179 | 3.05±0.008 | 3.5±0.014 | 3.66±0.028 | 3.75±0.028 | 3.9±0.014 | 4.2±0.028 | 4.5±0.070 |
| U1309 | 2.3±0.056 | 2.55±0.042 | 2.75±0.035 | 3.3±0.042 | 3.4±0.056 | 3.47±0.042 | 4.55±0.049 |
| U1360 | 3.42±0.070 | 3.85±0.049 | 4.1±0.091 | 4.4±0.091 | 4.8±0.063 | 5.1±0.077 | 5.6±0.049 |
